# 

**Published:** 1872-03

**Authors:** 


### CONTENTS:

SELECTIONS .............................. 129

ORIGINAL COMMUNICATIONS:

ARTICLE I. The Hypodermic Use of Corrosive Sublimate, in the Treatment of Syphilis. By Carl Proegler, M.D .............................. 162

ART. II. Disease Germs. (First paper.) By I. N. Danforth, M.D ........... 167

ART. III. A few Practical Hints on Sponge Tents and their Uses. (Second paper.) By Philip Adolphus, M.D .............................. 171

EDITORS' BOOK TABLE .............................. 177

EDITORIAL .............................. 180

ITEMS, NEWS AND GOSSIP .............................. 184

LOOT .............................. 186

